# Surgical and transcatheter aortic valve replacement align survival with general population expectations: insights from standardized mortality ratios

**DOI:** 10.3389/fcvm.2025.1547456

**Published:** 2025-04-11

**Authors:** Marin Boute, David De Azevedo, Christophe de Terwangne, Anne-Catherine Pouleur, Agnès Pasquet, Bernhard L. Gerber, Laurent de Kerchove, Christophe Beauloye, Joëlle Kefer, Frédéric Maes, Sophie Pierard, David Vancraeynest

**Affiliations:** ^1^ Pôle de Recherche Cardiovasculaire (CARD), Institut de Recherche Expérimentale et Clinique (IREC), Université Catholique de Louvain (UCLouvain), Brussels, Belgium; ^2^ Division of Cardiology, Cliniques Universitaires Saint-Luc, Brussels, Belgium; ^3^ Division of Geriatric Medicine, Cliniques Universitaires Saint-Luc, Brussels, Belgium; ^4^ Division of Cardiothoracic and Vascular Surgery, Cliniques Universitaires Saint-Luc, Brussels, Belgium; ^5^ Division of Cardiovascular Intensive Care, Cliniques Universitaires Saint-Luc, Brussels, Belgium

**Keywords:** TAVR, SAVR, general population, standardized mortality ratio, frailty, life expectancy, aortic stenosis, survival

## Abstract

**Background:**

Comparative long-term survival outcomes between transcatheter (TAVR) and surgical (SAVR) aortic valve replacement remain debated. While randomized controlled trials support TAVR's non-inferiority, real-world data indicate the opposite. Comparing SAVR and TAVR patients with matched reference populations may reduce bias from direct comparisons. We compared the 5-year overall survival rates of SAVR, non-frail TAVR, and frail TAVR patients with those of matched general population standards.

**Methods:**

All patients who underwent bioprosthetic SAVR or TAVR at a tertiary hospital from 2012 to 2021 were included. Based on intervention type and Clinical Frailty Scale, patients were divided into three groups: SAVR, non-frail TAVR, and frail TAVR. Survival was compared to individual-level age- and sex-matched general population data using standardized mortality ratios (SMRs).

**Results:**

The cohort included 939 SAVR, 328 non-frail TAVR, and 121 frail TAVR patients, with mean ages of 73.6, 85.3, and 85.6 years, and median EuroSCORE II values of 1.9%, 4.0%, and 5.2%, respectively. SAVR and non-frail TAVR patients had survival rates comparable to those of the reference population [SMR = 0.93 [0.76–1.14]; *p* = 0.437 and SMR = 0.94 [0.76–1.15]; *p* = 0.468]. Conversely, frail TAVR patients faced a 40% increased mortality risk compared with their reference population [SMR = 1.40 (1.04–1.88); *p* = 0.012].

**Conclusions:**

In non-frail patients, TAVR and SAVR both restore life expectancy to general population standards. For frail TAVR patients, the lower survival rate highlights frailty's important prognostic impact and underlines the ongoing challenge of refining patient selection to avoid futility.

## Graphical Abstract

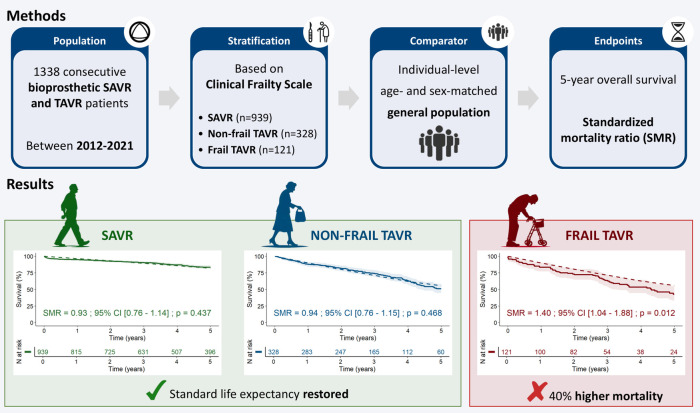

## 1 Introduction

Aortic stenosis is a major cardiovascular health challenge, with overall mortality rates remaining unchanged despite significant advances in its management over the last few decades (1). Currently, no pharmacological treatments have proven effective, leaving surgical (SAVR) or transcatheter (TAVR) aortic valve replacement as the primary options for managing this condition (2). Originally intended for high-risk patients, TAVR has since been extended to a wider range of candidates (3–5).

Randomized controlled trials (RCTs) have consistently demonstrated the non-inferiority of TAVR compared with SAVR across different risk profiles (6–12). However, result generalizability is often limited by strict inclusion criteria and controlled environments. In particular, frail patients are underrepresented, even within high-risk cohorts (6, 7, 13). On the other hand, observational studies capture real-world clinical practice more effectively, yielding insights complementing the controlled environments of RCTs (14). These studies often indicate that in real-world settings, TAVR may have worse outcomes than SAVR in the long term (15–17). Observational studies have their own limitations, particularly with respect to confounding factors. Propensity score matching (PSM), which is commonly used to balance observational cohorts, attempts to reduce biases but cannot account for unmeasured confounders (18). This limitation is particularly evident in studies comparing SAVR and TAVR, in which subjective clinical decisions and non-quantifiable factors often influence treatment decisions, potentially leading to biased results favoring SAVR (19). Furthermore, PSM often introduces selection bias by excluding unmatched subjects from the analysis. This issue is amplified in SAVR vs. TAVR studies, as young low-risk patients are typically directed toward SAVR, while older high-risk patients are more often considered for TAVR. Such a divide complicates matching and leads to the frequent exclusion of frail patients, who are predominantly assigned to TAVR. As a result, despite efforts to reflect clinical practice, observational studies are frequently scrutinized for reliability.

A practical approach to address these limitations is to compare dissimilar groups not directly with one another, but with respectively matched reference populations (20). This strategy mitigates poor cohort balancing and the selection bias introduced by excluding unmatched subjects. In this study, we will compare TAVR and SAVR patients' survival with that of matched general population standards, using up-to-date statistical methods (21). In addition, this study will specifically examine frail TAVR patients, who have often been excluded from previous analyses due to the constraints of PSM. By analyzing them as a distinct subgroup, we will explore the impact of frailty on the prognosis of patients treated with TAVR.

## 2 Materials and methods

### 2.1 Study population and design

**Figure 1:**
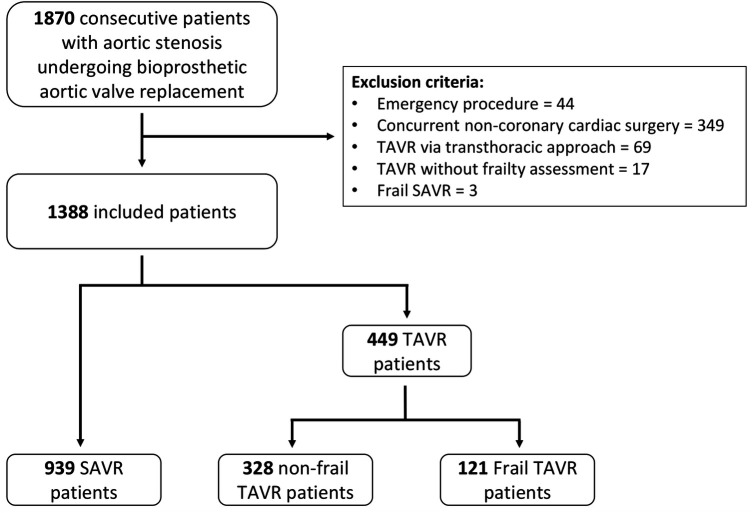
Flowchart illustrating patient selection and stratification into SAVR, non-frail TAVR, and frail TAVR groups. SAVR, surgical aortic valve replacement; TAVR, transcatheter aortic valve replacement*.*

This cohort study included all consecutive patients diagnosed with aortic stenosis who underwent bioprosthetic SAVR or TAVR at a tertiary care hospital between January 2012 and December 2021 (Figure 1). Exclusion criteria comprised: emergency procedures, additional cardiac surgeries beyond coronary artery bypass grafting, TAVR performed via transapical or transaortic approaches, and TAVR without a comprehensive pre-procedural geriatric assessment. Frail SAVR patients (*n* = 3) were excluded from the analysis due to insufficient sample size. A total of 1,388 patients were included in the analysis and divided into three groups based on geriatric frailty assessment: SAVR (*n* = 939), non-frail TAVR (*n* = 328), and frail TAVR (*n* = 121). Treatment decisions for SAVR vs. TAVR were guided by heart team discussions in accordance with guidelines. The primary endpoint was 5-year overall survival after intervention. Mortality data were obtained from the national healthcare system, with censoring applied at the last recorded interaction for patients without a documented death date. Baseline characteristics were collected at the time of intervention. Surgical risk was assessed through the European System for Cardiac Operative Risk Evaluation II (EuroSCORE II) (22). The study was conducted in accordance with institutional policies and the Declaration of Helsinki.

### 2.2 Frailty assessment

All aortic valve replacement candidates older than 75 years were first screened using the Identification of Seniors at Risk (ISAR) tool (23). Those with an ISAR score of ≥3, patients clinically needing further geriatric assessment, and all candidates for TAVR underwent a comprehensive geriatric assessment (CGA). The CGA, conducted by an experienced geriatric team, included evaluations of Basic and Instrumental Activities of Daily Living. Using Rockwood's validated classification tree, we derived the Clinical Frailty Scale (CFS) from these assessments (24). Patients with a CFS score ≥6 were classified as frail, in line with European Society of Cardiology guidelines (2).

### 2.3 Statistical analysis

Continuous variables were reported as means ± standard deviation or medians [interquartile range (IQR)], and categorical variables as frequencies and percentages. Appropriate tests (i.e., Student's *t*-test, Mann–Whitney *U* test, *χ*^2^ test, or Fisher's exact test) were applied based on distribution and expected frequencies. Survival curves were constructed using the Kaplan–Meier method and analyzed with log-rank tests. Propensity scores were estimated using a logistic regression model that included clinically and demographically relevant variables: age, male sex, body mass index, EuroSCORE II, dyspnea, angina, associated revascularization, history of coronary artery bypass grafting or percutaneous coronary intervention, history of myocardial infarction, history of cerebrovascular accident, peripheral arterial disease, atrial fibrillation, pacemaker implantation, neurological disorders, pulmonary disease, chronic kidney disease, arterial hypertension, diabetes, aortic valve area index, left ventricular ejection fraction, pulmonary hypertension, mitral regurgitation, and tricuspid regurgitation. Matching was performed using the nearest neighbor method with a caliper of 0.1 standard deviations of the logit propensity score. Covariate balance was assessed with standardized mean differences, with values <10% indicating satisfactory balance. Observed survival rates of the three patient groups were then compared to age- and sex-matched general population standards using standardized mortality ratios (SMRs) and one-sample log-rank tests (20). To achieve this, expected survival for each subgroup was derived from national actuarial tables (2009–2023) (25), ensuring individual-level matching based on age, sex, and observation year. Analyses were conducted using R version 4.2.1, with statistical significance set at *p* < 0.05.

## 3 Results

### 3.1 Baseline characteristics

The study included 1,388 patients: 939 underwent SAVR, and 449 underwent TAVR, with 328 (73.1%) classified as non-frail and 121 (26.9%) as frail. Baseline characteristics are summarized in Table 1. SAVR patients were younger (mean age 73.6 years) than TAVR patients (mean ages 85.3 and 85.6 years for non-frail and frail groups, respectively). The proportion of female patients increased across the groups, from 45.8% in SAVR to 51.4% in non-frail TAVR and 65.3% in frail TAVR. Operative risk, as reflected by EuroSCORE II, was lowest in SAVR patients (1.9%), followed by non-frail TAVR (4.0%) and frail TAVR (5.2%). When comparing SAVR to non-frail TAVR, notable differences were observed, with higher prevalence of cardiac, renal, and pulmonary comorbidities in non-frail TAVR patients. In contrast, non-frail and frail TAVR groups shared largely similar characteristics, except for a higher prevalence of severe dyspnea (82.6% vs. 61.3%) and prior cerebrovascular events (21.0% vs. 9.2%) in frail patients. Procedural characteristics, such as valve sizes, brands, and approaches, are detailed in Tables 2, 3. Notably, concurrent coronary artery bypass grafting was performed in 37.0% of SAVR patients, while 26.1% of TAVR patients underwent percutaneous coronary intervention within the past 6 months.

**Table 1:** Baseline characteristics of study participants.

Characteristic	Descriptives				SAVR vs. non-frail TAVR	Non-frail vs. frail TAVR
	Overall; *n* = 1;388	SAVR; *n* = 939	Non-frail TAVR; *n* = 328	Frail TAVR; *n* = 121	*p*-value	*p*-value
Clinical characteristics						
Age	77.4 ± 9.2	73.6 ± 8.0	85.3 ± 5.7	85.6 ± 5.4	**<0**.**001**	0.580
Male gender	752 (54.2%)	550 (58.6%)	160 (48.8%)	42 (34.7%)	**0**.**002**	**0**.**008**
Body Mass Index (kg/m^2^)	27.9 ± 5.3	28.5 ± 5.4	26.3 ± 4.6	27.9 ± 5.5	**<0**.**001**	**0**.**004**
EuroSCORE II (%)	2.4 (1.5–4.3)	1.9 (1.2–3.0)	4.0 (2.6–6.4)	5.2 (3.1–7.4)	**<0**.**001**	**0**.**007**
Dyspnea (NYHA ≥ III)	548 (39.5%)	247 (26.3%)	201 (61.3%)	100 (82.6%)	**<0**.**001**	**<0**.**001**
Angina (CCS ≥1)	379 (27.3%)	255 (27.2%)	94 (28.7%)	30 (24.8%)	0.600	0.416
Associated revascularization	463 (33.5%)	347 (37.0%)	80 (24.8%)	36 (29.8%)	**<0**.**001**	0.287
Medical history						
Previous Coronary Artery Bypass Graft	96 (6.9%)	41 (4.4%)	43 (13.1%)	12 (9.9%)	**<0**.**001**	0.360
Previous Percutaneous Coronary Intervention	267 (19.2%)	120 (12.8%)	108 (32.9%)	39 (32.2%)	**<0**.**001**	0.889
Previous Myocardial Infarction	160 (11.5%)	87 (9.3%)	61 (18.6%)	12 (9.9%)	**<0**.**001**	**0**.**027**
Previous Cerebrovascular Accident	84 (6.1%)	24 (2.6%)	29 (8.8%)	31 (25.6%)	**<0**.**001**	**<0**.**001**
Peripheral Arterial Disease	141 (10.2%)	62 (6.6%)	57 (17.4%)	22 (18.2%)	**<0**.**001**	0.843
Atrial Fibrillation	242 (17.4%)	104 (11.1%)	98 (29.9%)	40 (33.1%)	**<0**.**001**	0.517
Previous Pacemaker Implantation	119 (8.6%)	18 (1.9%)	74 (22.6%)	27 (22.3%)	**<0**.**001**	0.956
Pulmonary Disease	158 (11.4%)	60 (6.4%)	68 (20.7%)	30 (24.8%)	**<0**.**001**	0.355
Chronic Kidney Disease (eGFR ≤60 ml/min)	566 (40.8%)	298 (31.7%)	197 (60.1%)	71 (58.7%)	**<0**.**001**	0.791
Arterial Hypertension	1,098 (79.1%)	715 (76.1%)	275 (83.8%)	108 (89.3%)	**0**.**004**	0.151
Diabetes	338 (24.4%)	257 (27.4%)	58 (17.7%)	23 (19.0%)	**<0**.**001**	0.746
Echocardiographic findings						
Aortic Mean Gradient (mmHg)	47.3 ± 14.7	47.2 ± 15.3	47.7 ± 13.8	47.3 ± 12.2	0.638	0.798
Aortic Valve Area Index (cm^2^/m^2^)	0.39 ± 0.10	0.40 ± 0.10	0.38 ± 0.08	0.36 ± 0.09	**<0**.**001**	0.212
Left Ventricular Ejection Fraction (%)	62.5 ± 14.1	63.8 ± 13.4	59.6 ± 15.1	60.4 ± 15.7	**<0**.**001**	0.646
Pulmonary Hypertension (PASP ≥45 mmHg)	381 (27.4%)	190 (20.2%)	138 (42.1%)	53 (43.8%)	**<0**.**001**	0.742
Mitral Regurgitation (mild/moderate)	40 (2.9%)	12 (1.3%)	20 (6.2%)	8 (6.6%)	**<0**.**001**	0.865
Tricuspid Regurgitation (mild/moderate)	73 (5.3%)	24 (2.6%)	37 (11.4%)	12 (9.9%)	**<0**.**001**	0.652

CCS, canadian cardiovascular society score; eGFR, estimated glomerular filtration rate by CKD-EPI formula; EuroSCORE II, European system for cardiac operative risk evaluation II; NYHA, New York heart association score; PASP, pulmonary artery systolic pressure; SAVR, surgical aortic valve replacement; TAVR, transcatheter aortic valve replacement.

*P*-values <0.10 are highlighted in bold.

**Table 2:** Procedural characteristics of SAVR cohort.

Characteristic	SAVR; *n* = 939
Bioprosthetic valve	939 (100.0%)
Valve Brand	
Avalus	15 (1.6%)
Crown	18 (1.9%)
Edwards	527 (56.1%)
Hancock II	113 (12.0%)
Trifecta	266 (28.3%)
Valve Size	
19	7 (0.7%)
21	115 (12.2%)
23	319 (34.0%)
25	315 (33.5%)
27	163 (17.4%)
29	20 (2.1%)
True Internal Diameter (mm)	21.9 ± 2.0
Surgical Approach Route	
Full Sternotomy	780 (83.1%)
Mini-Sternotomy	159 (16.9%)
Associated CABG (during index procedure)	347 (37.0%)

CABG, coronary artery bypass grafting; SAVR, surgical aortic valve replacement*.*

**Table 3:** Procedural characteristics of TAVR cohorts.

Characteristic	Overall; *n* = 449	Non-frail TAVR; *n* = 328	Frail TAVR; *n* = 121	*p*-value
Valve Brand				**0** **.** **084**
CoreValve	74 (16.5%)	47 (14.3%)	27 (22.3%)	
Evolut R	262 (58.4%)	202 (61.6%)	60 (49.6%)	
Portico	26 (5.8%)	21 (6.4%)	5 (4.1%)	
Sapien 3	22 (4.9%)	15 (4.6%)	7 (5.8%)	
Sapien XT	65 (14.5%)	43 (13.1%)	22 (18.2%)	
Valve Size				0.394
23	39 (8.7%)	30 (9.1%)	9 (7.4%)	
25	8 (1.8%)	6 (1.8%)	2 (1.7%)	
26	133 (29.6%)	90 (27.4%)	43 (35.5%)	
27	6 (1.3%)	6 (1.8%)	0 (0.0%)	
29	162 (36.1%)	117 (35.7%)	45 (37.2%)	
31	13 (2.9%)	9 (2.7%)	4 (3.3%)	
34	88 (19.6%)	70 (21.3%)	18 (14.9%)	
True Internal Diameter (mm)	24.2 ± 2.6	24.3 ± 2.6	24.1 ± 2.4	0.422
Transcatheter Approach Route				0.570
Transcarotidian	35 (7.8%)	27 (8.2%)	8 (6.6%)	
Transfemoral	414 (92.2%)	301 (91.8%)	113 (93.4%)	
Associated PCI (<6 months before TAVR)	116 (26.1%)	80 (24.8%)	36 (29.8%)	0.287

PCI, percutaneous coronary intervention; TAVR, transcatheter aortic valve replacement*.*

*P*-values <0.10 are highlighted in bold.

### 3.2 Unadjusted survival

**Figure 2:**
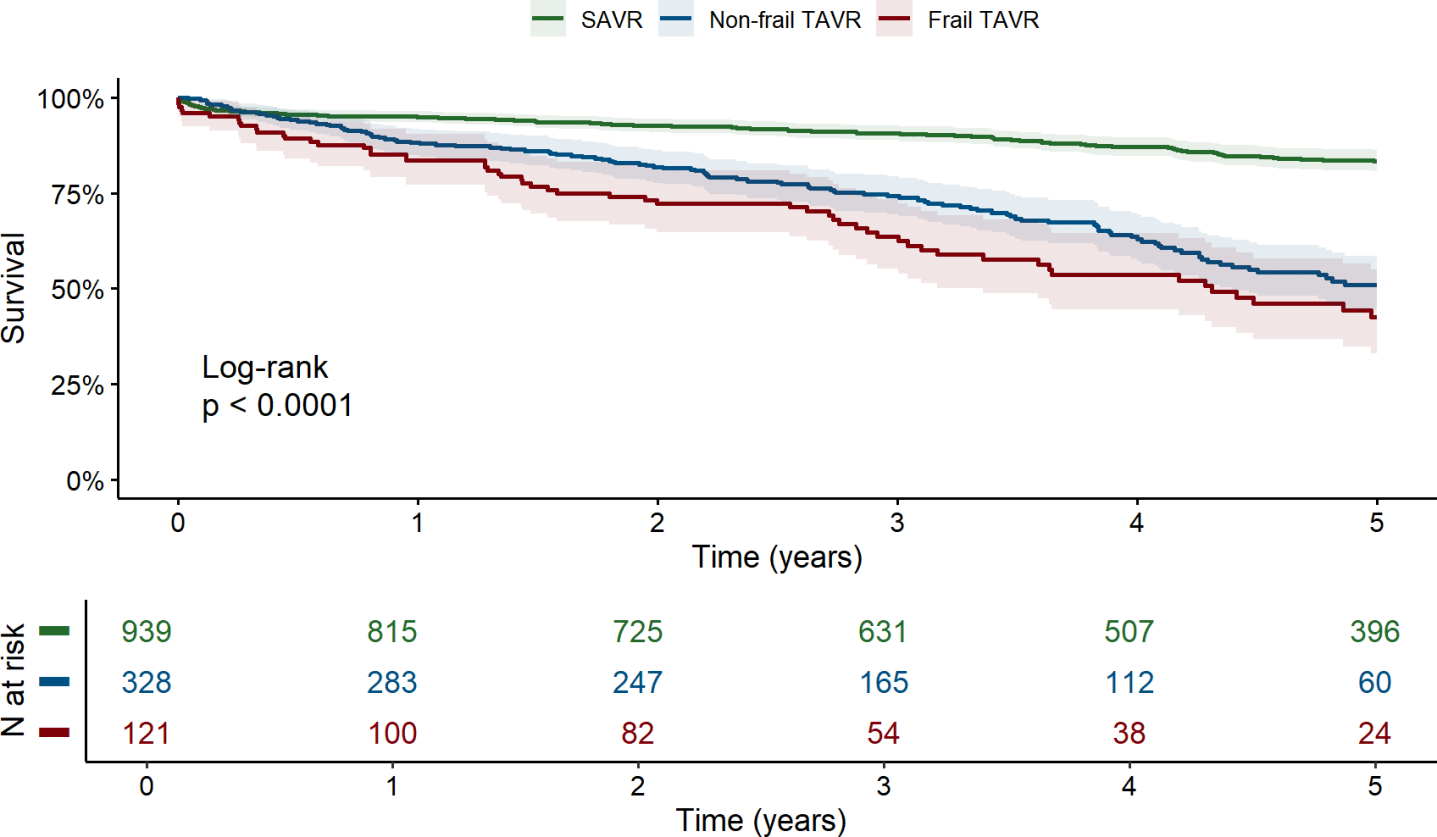
Unadjusted kaplan–meier survival curves for SAVR, non-frail TAVR, and frail TAVR patients (with 95% confidence intervals). SAVR, surgical aortic valve replacement; TAVR, transcatheter aortic valve replacement.

Over a median follow-up of 50.4 months (IQR: 31.0–82.9 months), 297 deaths (21.4%) were recorded in the cohort: 122 (13.0%) in SAVR, 119 (36.3%) in non-frail TAVR, and 56 (46.3%) in frail TAVR group. Significant differences in 5-year survival rates were observed among SAVR, non-frail TAVR, and frail TAVR patients (83.4% vs. 51.1% vs. 42.6%, respectively, *p* < 0.001, Figure 2).

### 3.3 Propensity-matched analysis between SAVR and non-frail TAVR

**Figure 3:**
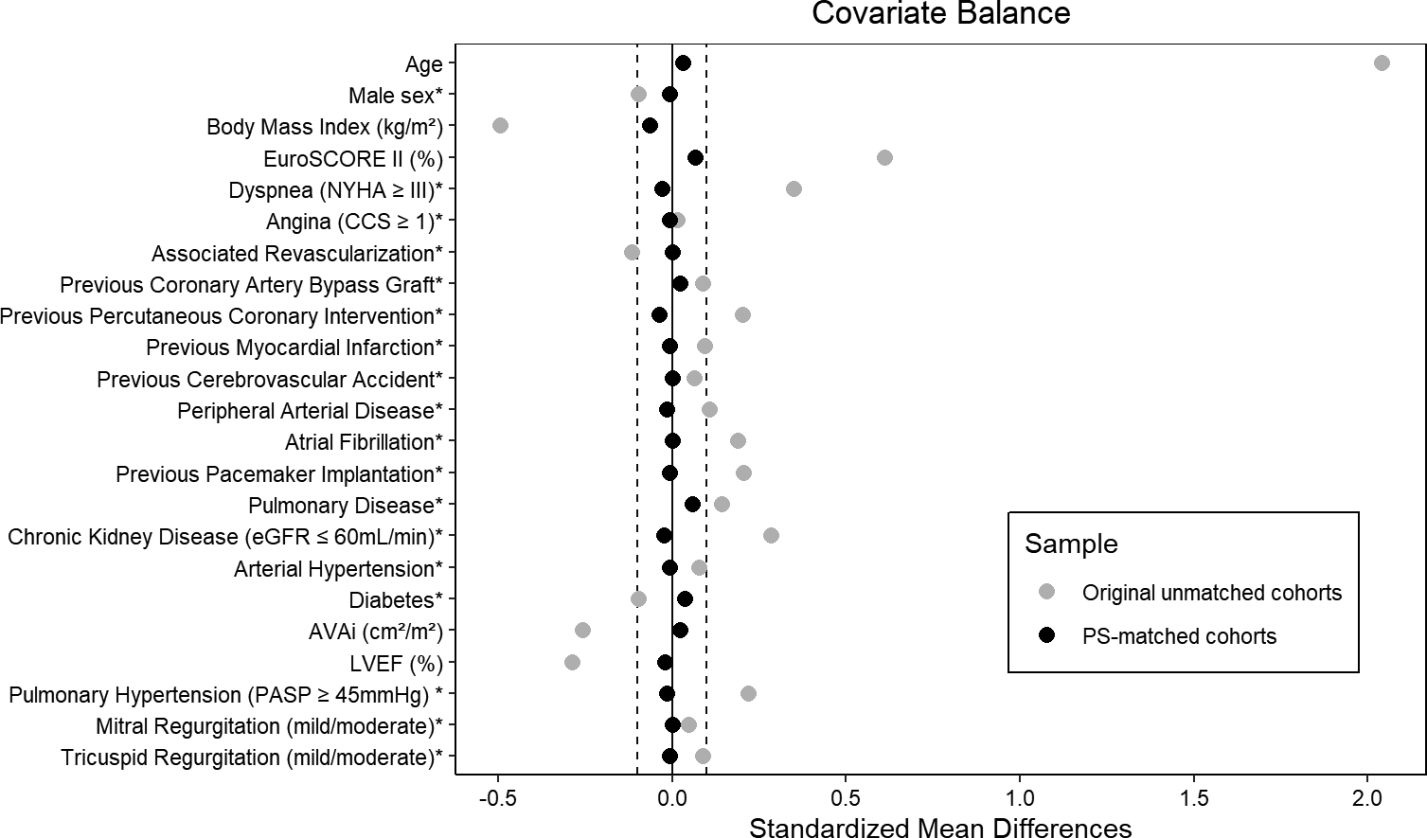
Covariate balance assessment before and after propensity score matching. *For binary variables, the displayed value represents the difference in proportions. AVAi, aortic valve area index; CCS, canadian cardiovascular society score; eGFR, estimated glomerular filtration rate by CKD-EPI formula; EuroSCORE II, European system for cardiac operative risk evaluation II; LVEF, left ventricular ejection fraction; NYHA, New York heart association score; PASP, pulmonary artery systolic pressure; PS, propensity score.

**Figure 4:**
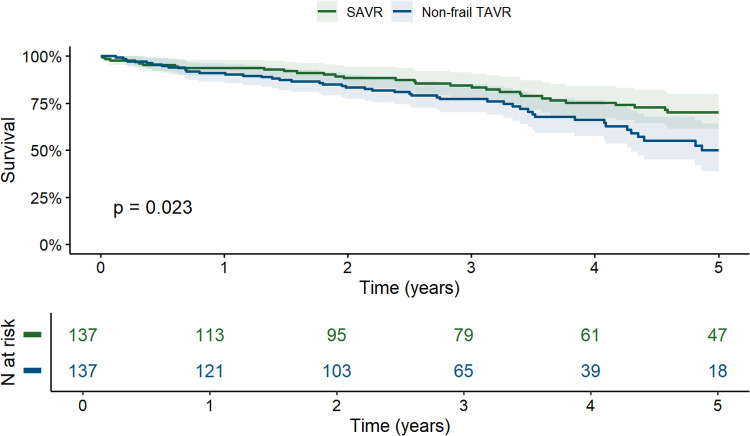
Kaplan–meier survival curves for the propensity score-matched SAVR and non-frail TAVR cohorts (with 95% confidence intervals). SAVR, surgical aortic valve replacement; TAVR, transcatheter aortic valve replacement.

Propensity score matching produced 137 matched pairs, balancing demographic and clinical characteristics effectively between SAVR and non-frail TAVR groups (Table 4), with standardized mean differences within the prespecified range (Figure 3). The matched cohort (mean age 81.8 years, 51.5% female, median EuroSCORE II 2.8%) aligned more closely with the baseline characteristics of the original non-frail TAVR group, particularly in age, sex, and comorbidities like atrial fibrillation and pulmonary hypertension. Post-matching, 5-year survival remained significantly higher in the SAVR cohort (70.1%) than in the non-frail TAVR cohort (49.9%), indicating notable differences in long-term outcomes (HR = 1.71, 95% CI: 1.07–2.73; *p* = 0.023; Figure 4).

**Table 4:** Baseline characteristics of selected SAVR and non-frail TAVR patients after propensity score matching.

Characteristic	SAVR; *n* = 137	Non-frail TAVR; *n* = 137	*p*-value
Clinical characteristics			
Age	81.7 ± 4.9	81.8 ± 6.0	0.790
Male gender	67 (48.9%)	66 (48.2%)	0.904
Body Mass Index (kg/m^2^)	27.3 ± 4.6	27.0 ± 5.0	0.616
EuroSCORE II (%)	2.5 (1.9–4.4)	3.0 (2.1–4.9)	0.227
Dyspnea (NYHA ≥ III)	66 (48.2%)	62 (45.3%)	0.628
Angina (CCS ≥1)	29 (21.2%)	28 (20.4%)	0.882
Associated revascularization	29 (21.2%)	27 (20.1%)	0.836
Medical history			
Previous Coronary Artery Bypass Graft	12 (8.8%)	15 (10.9%)	0.543
Previous Percutaneous Coronary Intervention	32 (23.4%)	27 (19.7%)	0.462
Previous Myocardial Infarction	16 (11.7%)	15 (10.9%)	0.849
Previous Cerebrovascular Accident	6 (4.4%)	6 (4.4%)	>0.999
Peripheral Arterial Disease	15 (10.9%)	13 (9.5%)	0.690
Atrial Fibrillation	34 (24.8%)	34 (24.8%)	>0.999
Previous Pacemaker Implantation	10 (7.3%)	9 (6.6%)	0.812
Pulmonary Disease	14 (10.2%)	22 (16.1%)	0.153
Chronic Kidney Disease (eGFR ≤60 ml/min)	73 (53.3%)	70 (51.1%)	0.717
Arterial Hypertension	115 (83.9%)	114 (83.2%)	0.870
Diabetes	23 (16.8%)	28 (20.4%)	0.438
Echocardiographic findings			
Aortic Mean Gradient (mmHg)	48.0 ± 16.6	48.0 ± 11.2	0.992
Aortic Valve Area Index (cm^2^/m^2^)	0.38 ± 0.09	0.38 ± 0.08	0.877
Left Ventricular Ejection Fraction (%)	62.4 ± 12.6	62.1 ± 13.0	0.805
Pulmonary Hypertension (PASP ≥45 mmHg)	50 (36.5%)	48 (35.0%)	0.801
Mitral Regurgitation (mild/moderate)	6 (4.4%)	6 (4.4%)	0.990
Tricuspid Regurgitation (mild/moderate)	10 (7.3%)	9 (6.6%)	0.825

CCS, canadian cardiovascular society score; eGFR, estimated glomerular filtration rate by CKD-EPI formula; EuroSCORE II, European system for cardiac operative risk evaluation II; NYHA, New York heart association score; PASP, pulmonary artery systolic pressure; SAVR, surgical aortic valve replacement; TAVR, transcatheter aortic valve replacement*.*

### 3.4 Observed vs. expected survival analysis—standardized mortality ratios

**Figure 5:**
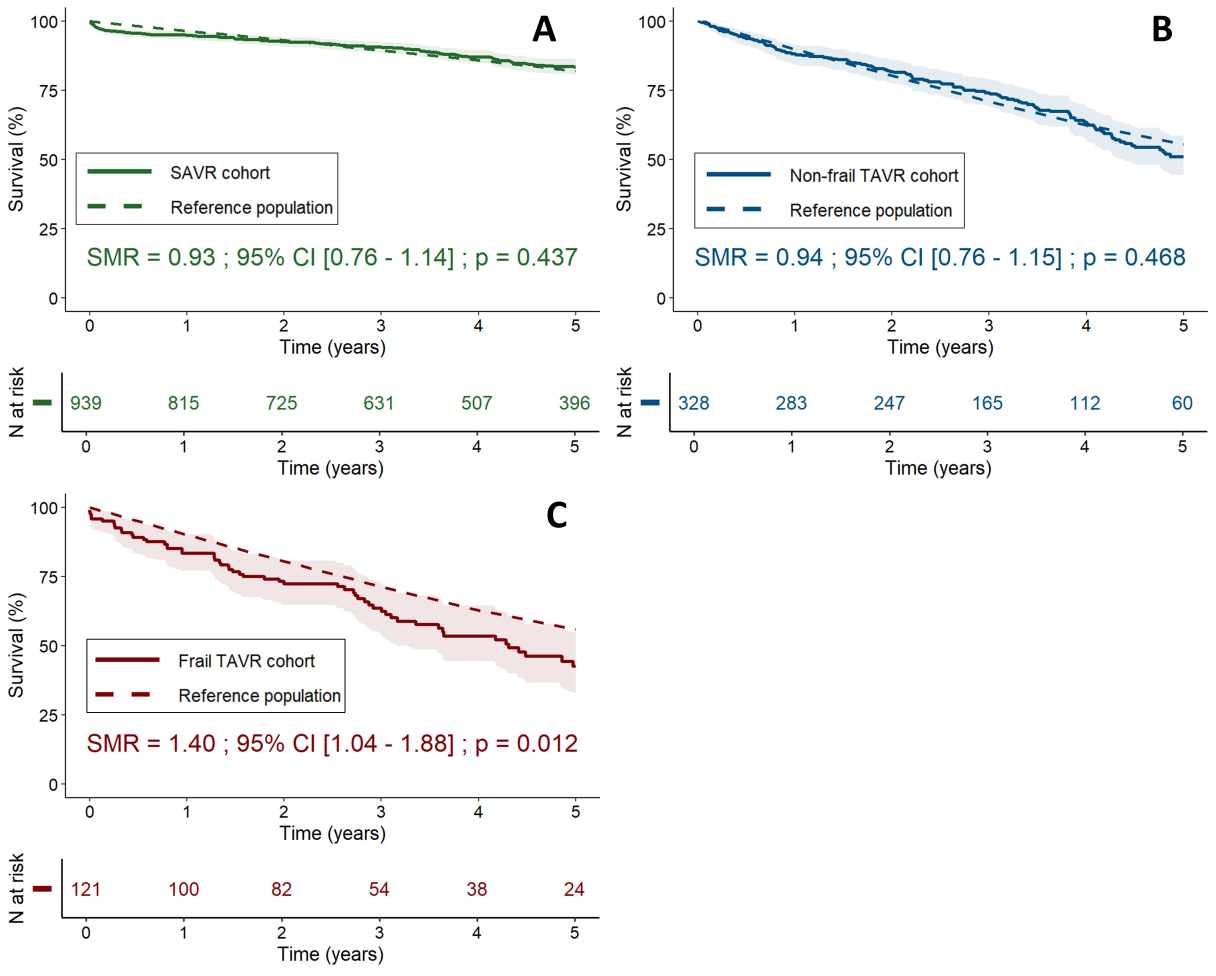
Standardized mortality ratios for SAVR **(A)**, non-frail TAVR **(B)**, and frail TAVR **(C)** patients. Observed survival curves (solid lines, with 95% confidence intervals) are compared with expected survival (dashed lines) based on individual-level age- and sex-matched general population data. CI, confidence interval; SAVR, surgical aortic valve replacement; SMR, standardized mortality ratio; TAVR, transcatheter aortic valve replacement.

We used SMRs to assess whether observed death counts in each patient group exceeded those expected based on standard population mortality rates. SAVR patients showed no significant survival difference compared with their respective age- and sex-matched general population [SMR = 0.93; 95% CI (0.76–1.14); *p* = 0.437, Figure 5A], nor did non-frail TAVR patients [SMR = 0.94; 95% CI (0.76–1.15); *p* = 0.468, Figure 5B]. In contrast, frail TAVR patients displayed significantly higher mortality than their reference population, with a 40% increased mortality risk [SMR = 1.40; 95% CI (1.04–1.88); *p* = 0.012, Figure 5C].

## 4 Discussion

This study compared the outcomes of SAVR and TAVR patients. In addition to a direct comparison, we assessed their outcomes against reference populations using SMR, a robust statistical technique that enables individual-level matched comparisons with general population data, providing new insights into survival results in this context (20). Given the significant disparity in frailty distribution between treatment groups, we strategically divided TAVR patients into frail and non-frail subgroups. Our analysis demonstrated that survival rates for both SAVR and non-frail TAVR groups were close to expected rates for similar demographic groups in the general population, suggesting no inherent survival disadvantage with these interventions. However, frail TAVR patients experienced significantly lower survival (a 40% increased mortality risk over 5 years) compared with their reference population, highlighting frailty as a pivotal factor influencing outcomes (Graphical abstract).

### 4.1 Postoperative prognosis in aortic valve replacement: bridging the gap between RCT and observational studies

Compared with RCTs, the composition of our SAVR cohort closely resembled the low-risk populations described in PARTNER 3 and Evolut Low Risk trials (10, 11). Conversely, our TAVR patients were more aligned with the high-risk groups observed in PARTNER 1 and CoreValve High Risk (6, 7). However, the proportion of frailty in our TAVR cohort (27%) exceeded the 16% and 6%–11% ranges reported in these trials, respectively, reflecting a more inclusive approach to patient selection in real-world settings compared with the controlled environments of RCTs (16, 26, 27). However, the older age profile of our cohort contrasts with the emerging use of TAVR among younger populations, as evidenced by the 5-year age gap with U.S. counterparts (4). While our results provide valuable insights into Europe's current predominant population receiving TAVR, extrapolating these findings to younger, less comorbid populations requires careful consideration. Future research should focus on these younger populations through comparative analyses with age-matched general populations to better understand the evolving implications of TAVR's expanding application.

RCTs consistently demonstrate the non-inferiority of TAVR in controlled settings, yet real-world studies often suggest a potential survival penalty when treating more heterogeneous aortic stenosis populations with TAVR. This discrepancy may partly arise from broader patient selection in observational studies but also reflects potential methodological biases. Despite its common use, PSM frequently suffers from inadequate application that can lead to statistical shortcomings (19). Proper matching requires including all observable covariates relevant to potential outcomes and ensuring patients have a realistic probability of receiving either treatment. These criteria are often unmet in SAVR vs. TAVR studies conducted in clinical practice, where treatment selection is influenced by subjective or intangible factors, and TAVR patients are frequently ineligible for SAVR. To address these challenges, our study benchmarked survival outcomes against demographically matched general populations. This approach preserves the real-world composition of the study cohorts and avoids the extensive adjustments required for direct comparisons, which can distort findings. Unlike earlier observational studies, this approach demonstrated no inherent survival disadvantage with either intervention, showing that both SAVR and non-frail TAVR restore life expectancy. By reproducing prior findings of TAVR inferiority through PSM and contrasting them with general population benchmarks, we identified methodological bias as a likely contributor to discrepancies in earlier studies, rather than true differences in treatment efficacy. These findings underscore the importance of complementary approaches, such as general population benchmarking, to minimize reliance on a single method and provide a broader perspective on treatment outcomes.

### 4.2 Prognostic implication of frailty in TAVR

Our study underscores the fact that while non-frail patients typically see their life expectancy normalized following aortic valve replacement, this restoration appears incomplete for frail patients for whom TAVR is proposed. However, because we can expect that these patients have a much higher burden of comorbidities and frailty index than their matched general population counterparts, our finding mainly reflects the adverse prognostic impact of frailty rather than an absence of TAVR benefit in frail patients. In fact, our data show significantly higher 1- and 2-year survival rates (83.5% and 72.4%) compared with the medical therapy arm of the PARTNER B trial (49.3% at 1 year and 32.4% at 2 years), demonstrating that TAVR confers a survival advantage even in frail patients (28). Furthermore, even though our study did not directly assess quality of life, existing literature suggests that, in this regard, TAVR offers significant improvements, which should be factored into evaluations of its overall benefit (29).

While TAVR confers survival advantages, it does not fully address the broader prognostic determinants of frailty, including comorbidities and increased adverse events. As such, the inability to completely align frail TAVR patients' survival with that of the general population raises important questions about what constitutes an “acceptable survival gap” to justify the procedure, particularly when balanced against quality-of-life improvements and patient preferences. It also underscores concerns about potential futility in certain cases. Despite widespread agreement on the need to avoid futile interventions, there is still ambiguity around how best to define and predict futility in TAVR (30). Current predictive models aimed at identifying futile interventions show only moderate accuracy, limiting their implementation in clinical practice (31). Future advancements, particularly in machine learning, hold promise for improving predictive accuracy by integrating complex, multidimensional data that traditional models cannot efficiently manage. However, shared decision-making through Heart Teams remains indispensable for addressing non-quantifiable factors such as patient preferences, social support, and ethical considerations. Overall, frailty should not contraindicate TAVR outright but should prompt individualized decision-making that balances survival with quality-of-life improvements and patient-centered outcomes. Future research will be essential to clarify which subset of frail TAVR patients stands to gain the most meaningful benefit. These efforts will help advance our ability to define and avoid truly futile interventions in this vulnerable population.

### 4.3 Limitations

This study's single-center design limits the generalizability of its findings. However, the alignment of our baseline characteristics with larger contemporary studies and the consistency of our PSM results with existing literature suggest that the observed treatment effects are not solely influenced by local practice patterns. Nevertheless, a cohort effect remains possible, as our predominantly elderly population may not fully reflect broader clinical contexts. The observational design of this study inherently carries limitations. However, it allows for the capture of real-world conditions, which randomized controlled trials often cannot replicate. Using the general population as the reference group, rather than strictly healthy individuals, could also be seen as a limitation. Nevertheless, this approach provides a realistic benchmark by capturing how patients fare against the backdrop of normal aging and common comorbidities, aligning with the primary objective of this study. It would also have been interesting to compare our frail TAVR cohort to a similarly frail population without aortic stenosis to see whether TAVR restores life expectancy in frail aortic stenosis patients to levels expected for frail individuals in general. Unfortunately, we did not have access to such a reference population to carry out this analysis. Finally, the binary classification of frailty in this study simplifies a complex and multifactorial condition. While this approach enables statistical analysis, it may not translate well into clinical practice, where the full spectrum of frailty influences clinical decision-making and patient prognosis.

## 5 Conclusion

In our study, both TAVR and SAVR can restore life expectancy to levels comparable with those of a matched reference population in non-frail aortic stenosis patients. However, the significantly lower survival rate observed in frail TAVR patients highlights the need for careful patient selection and the development of tailored treatment strategies considering both benefits and limitations of TAVR in this vulnerable population. Further prospective studies are needed to refine the selection criteria for TAVR in frail patients, ensuring that treatment decisions are aligned with both survival and quality of life outcomes.

## Data Availability

The raw data supporting the conclusions of this article will be made available by the authors, without undue reservation.
